# Higher O-GlcNAc Levels Are Associated with Defects in Progenitor Proliferation and Premature Neuronal Differentiation during *in-Vitro* Human Embryonic Cortical Neurogenesis

**DOI:** 10.3389/fncel.2017.00415

**Published:** 2017-12-21

**Authors:** Shama Parween, Divya S. Varghese, Mustafa T. Ardah, Ashok D. Prabakaran, Eric Mensah-Brown, Bright Starling Emerald, Suraiya A. Ansari

**Affiliations:** ^1^Department of Biochemistry, College of Medicine and Health Sciences, United Arab Emirates University, Al Ain, United Arab Emirates; ^2^Department of Anatomy, College of Medicine and Health Sciences, United Arab Emirates University, Al Ain, United Arab Emirates

**Keywords:** hESCs, pluripotency, cortical neurogenesis, O-GlcNAcylation, cell proliferation and differentiation, neurodevelopment

## Abstract

The nutrient responsive O-GlcNAcylation is a dynamic post-translational protein modification found on several nucleocytoplasmic proteins. Previous studies have suggested that hyperglycemia induces the levels of total O-GlcNAcylation inside the cells. Hyperglycemia mediated increase in protein O-GlcNAcylation has been shown to be responsible for various pathologies including insulin resistance and Alzheimer's disease. Since maternal hyperglycemia during pregnancy is associated with adverse neurodevelopmental outcomes in the offspring, it is intriguing to identify the effect of increased protein O-GlcNAcylation on embryonic neurogenesis. Herein using human embryonic stem cells (hESCs) as model, we show that increased levels of total O-GlcNAc is associated with decreased neural progenitor proliferation and premature differentiation of cortical neurons, reduced AKT phosphorylation, increased apoptosis and defects in the expression of various regulators of embryonic corticogenesis. As defects in proliferation and differentiation during neurodevelopment are common features of various neurodevelopmental disorders, increased O-GlcNAcylation could be one mechanism responsible for defective neurodevelopmental outcomes in metabolically compromised pregnancies such as diabetes.

## Introduction

Maternal hyperglycemia has long been implicated with adverse neurodevelopmental outcomes in the offspring (Krakowiak et al., 2012; Lopaczynski, 2012; Xu et al., 2014a; Ornoy et al., 2015). Various epidemiological studies as well as studies using animal models of maternal diabetes have suggested a link between the two (Kinney et al., 2003; Hami et al., 2015). Although these studies established a connection between maternal hyperglycemia and neurodevelopmental disorders in children, the molecular mechanism/s which are responsible for this association have not been established, especially in humans. There were some studies which indicated that, genetics and environmental factors may be playing critical roles in the outcome of these disorders (van Loo and Martens, 2007; Van Winkel et al., 2010). One molecular mechanism through which uncontrolled hyperglycemia could exert its effect on cellular metabolism is proposed to be through O-GlcNAc modification of proteins. Increase in blood glucose levels are shown to increase the levels of O-GlcNAcaylation through hexosamine biosynthesis pathway (HBP). Glucose enters the HBP pathway as fructose-6-phosphate and through several steps is converted to the end product UDP-GlcNAc. UDP-GlcNAc is the substrate for protein O-GlcNAcylation at serine/threonine amino acids of specific nucleocytoplasmic proteins, catalyzed by enzyme, β-linked-O-GlcNAc transferase (OGT). Enzyme, β-N-acetylglucosaminidase (OGA) catalyzes the reverse reaction of removing this modification (Love and Hanover, 2005; Hart et al., 2007). Thus, O-GlcNAcylation is a dynamic protein modification similar to protein phosphorylation where O-GlcNAc moiety is dynamically deposited and removed from the proteins with the help of the enzymes, OGT/OGA (Akimoto et al., 2005; Vaidyanathan et al., 2014). Increased O-GlcNAc levels have been reported in mothers with uncontrolled diabetes as well as in cell cultures with high glucose (25 mM or higher) (Harwood and Hanover, 2014; Bond and Hanover, 2015) suggesting that the activation of HBP pathway and the dynamic OGlcNAc protein modification play important roles in various cellular metabolic processes (Zachara and Hart, 2006).

It has been proposed that Protein O-GlcNAcylation regulates cellular metabolism by different mechanisms (Ruan et al., 2013) such as post-translational O-GlcNAcylation of signaling proteins and transcription factors (Issad and Kuo, 2008; Ozcan et al., 2010; Ma and Hart, 2014), O-GlcNAc modification of histone and gene expression (Fujiki et al., 2011; Xu et al., 2014b), O-GlcNAcylation of conserved C-terminal domain (CTD) of RNA Pol II affects transcription (Comer and Hart, 2001; Ranuncolo et al., 2012). While hyperglycemia leads to increased O-GlcNAcylation of several proteins, it often occurs on the same or adjacent phosphorylation sites and thus the interplay between these two modifications may have roles in the regulation of downstream signaling pathways (Wells et al., 2003). OGT/OGA, the enzyme pair responsible for the addition or removal of O-GlcNAc modification is highly expressed in the brain suggesting that protein O-GlcNAcylation may have a role in the normal development and differentiation of central nervous system (CNS). At the cellular level, increased O-GlcNAc levels induce neuronal apoptosis (Shi et al., 2015). Among the various other mechanisms, dynamic interplay of post-translational O-GlcNAcylation and phosphorylation of specific proteins is known to be responsible for cellular apoptosis in the brain (Liu et al., 2004; Zhu et al., 2015; Ning et al., 2017).

Recent studies have established a clear role for O-GlcNAc modification of proteins in the diseases of central nervous system including Parkinson's and Alzheimer's disease (Wani et al., 2016). Several proteins which are involved in the etiology of Alzheimer's disease such as Tau, beta-amyloid precursor protein, and synaptosomal proteins are found to be O-GlcNAcylated (Wang et al., 2016).

However, the effect of O-GlcNAc levels in neurodevelopment is not well studied especially in human. We hypothesized that uncontrolled maternal hyperglycemia with increase in O-GlcNAc levels in the developing embryo may impair developmental neurogenesis. In order to study this correlation at the cellular and molecular level, we employed hESC's differentiation toward cortical neurons using protocols published by us and others (Chambers et al., 2009; Shi et al., 2012a; van de Leemput et al., 2014; Basanta-Sanchez et al., 2016). As many neurodevelopmental disorders involve defects in cell proliferation and differentiation during development (Gigek et al., 2015; Homem et al., 2015), they could be recapitulated during proliferation and differentiation of hESCs toward neuronal fate (Ernst, 2016). We herein show that higher O-GlcNAc levels result in reduced cell proliferation and premature cortical neurogenesis due to altered expression of various effectors of cortical neurogenesis.

## Results

As hyperglycemia increases the levels of total O-GlcNAc and the enzymes responsible for O-GlcNAc modification are also expressed highly in the brain, we hypothesized that maternal hyperglycemia could chronically increase the levels of total O-GlcNAc inside the developing brain of the embryo. To verify this, we used Wistar rats as model system and treated them with streptozotocin (STZ) to induce hyperglycemia. We then used embryos of STZ treated hyperglycemic and control animals to check the levels of total O-GlcNAc in the developing brains (E16.5 and E18.5) by western blotting. Our results show that the levels of total O-GlcNAc were substantially high in the brains of embryos from hyperglycemic rats (Figure S1). This result suggested that maternal hyperglycemia during pregnancy indeed increases the levels of total O-GlcNAc inside the developing brain of the embryo.

**Figure 1:**
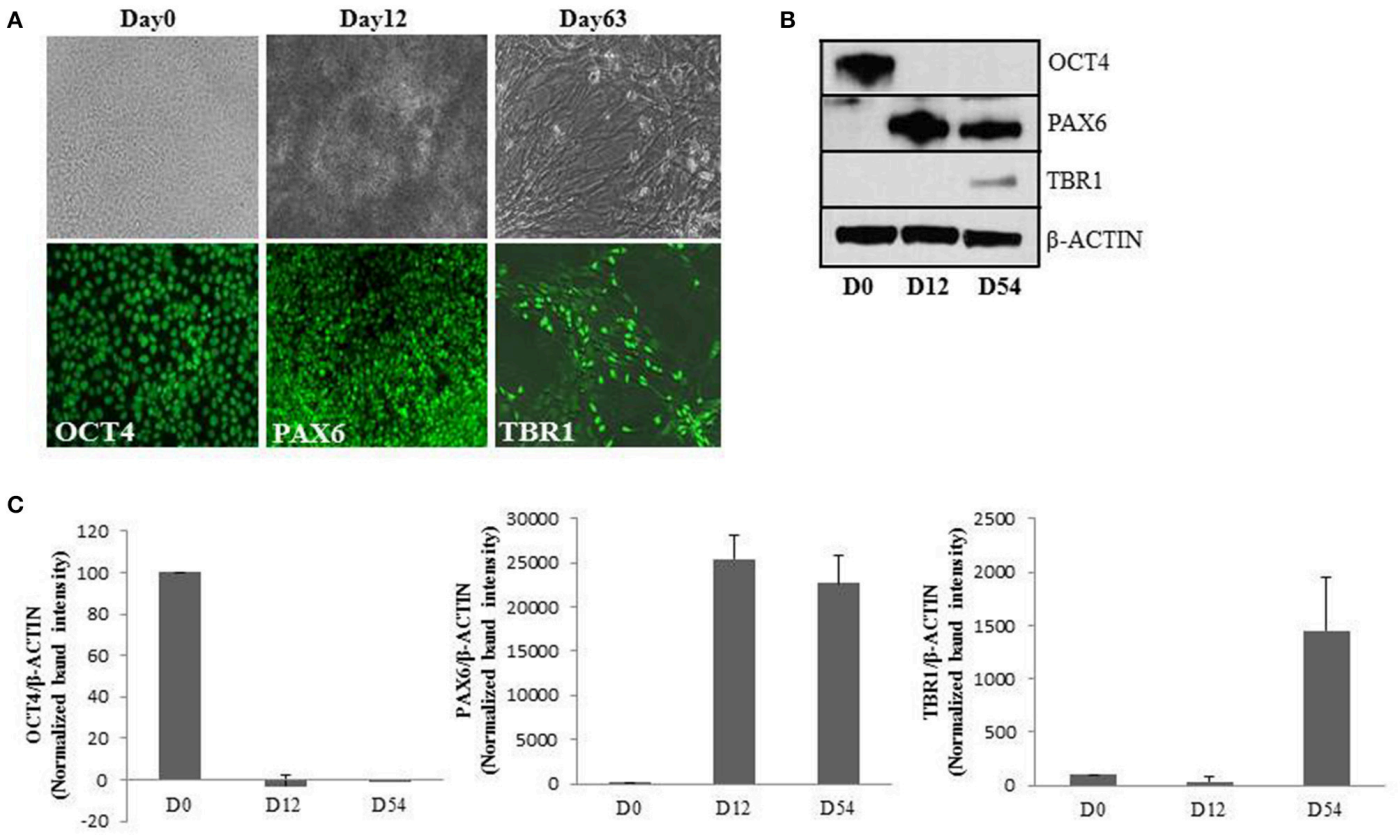
Establishment of *in-vitro* cortical neurogenesis. **(A)** Immunocytochemical analysis of undifferentiated H9 cells (Day0), neural progenitors (Day12) and early born deep layer cortical neurons (Day63). Top panel indicates bright-field images and the bottom panel shows immunostaining using anti OCT4, PAX6, and TBR1 antibodies (scale bar = 100 μm). **(B)** Western blot analysis of OCT4, PAX6, and TBR1 from lysates collected at D0, D12, and D54 of cortical neurogenesis. The expression of β-*ACTIN* was used as loading control. The data are representative of three independent biological replicates. **(C)** Densitometric quantitation of western blots from panel **(B)**. The values are expressed as percentage, relative to the values for Day0 samples set as 100%. The data are represented as mean ± standard deviation.

Therefore, we proceeded to verify the effect of chronically elevated O-GlcNAc on embryonic neurogenesis in human. We used human embryonic stem cells (hESCs) as a model and used our established protocol to recapitulate the key events of embryonic cortical neurogenesis at cellular and molecular level (Mariani et al., 2012; Shi et al., 2012b). We differentiated H9 hESCs toward neural fate for 12 days to obtain neural progenitor cells (Chambers et al., 2009). Upon day12 of neural induction, these progenitor cells were differentiated into neurons of cortical fate by culturing for another 40–50 days (van de Leemput et al., 2014; Basanta-Sanchez et al., 2016). We confirmed the differentiation process by using lineage specific markers during the course of differentiation by immunocytochemistry and western blotting. We observed high levels of expression of OCT4, PAX6, and TBR1 by immuno-staining at day0, day12, and day63 of neural induction respectively (Figure 1A). Western blotting further confirmed high levels of OCT4 expression at day0 (pluripotent) of neural induction which was significantly downregulated from day12 onwards whereas expression of neural progenitor marker, PAX6 was elevated from day12. Early born cortical neuron specific marker, TBR1 was detected on day54 (Figures 1B,C). Several other markers of cortical neurogenesis which express in stage specific manner were tested at different stages of neural induction as well (data not shown). Together, these results confirmed the feasibility of the cortical neurogenesis protocol and validated our hESC model system which we used to understand the effect of elevated O-GlcNAc levels on neuronal differentiation.

### Effect of elevated O-GlcNAc levels on neural progenitor differentiation of hESCs

**Figure 2:**
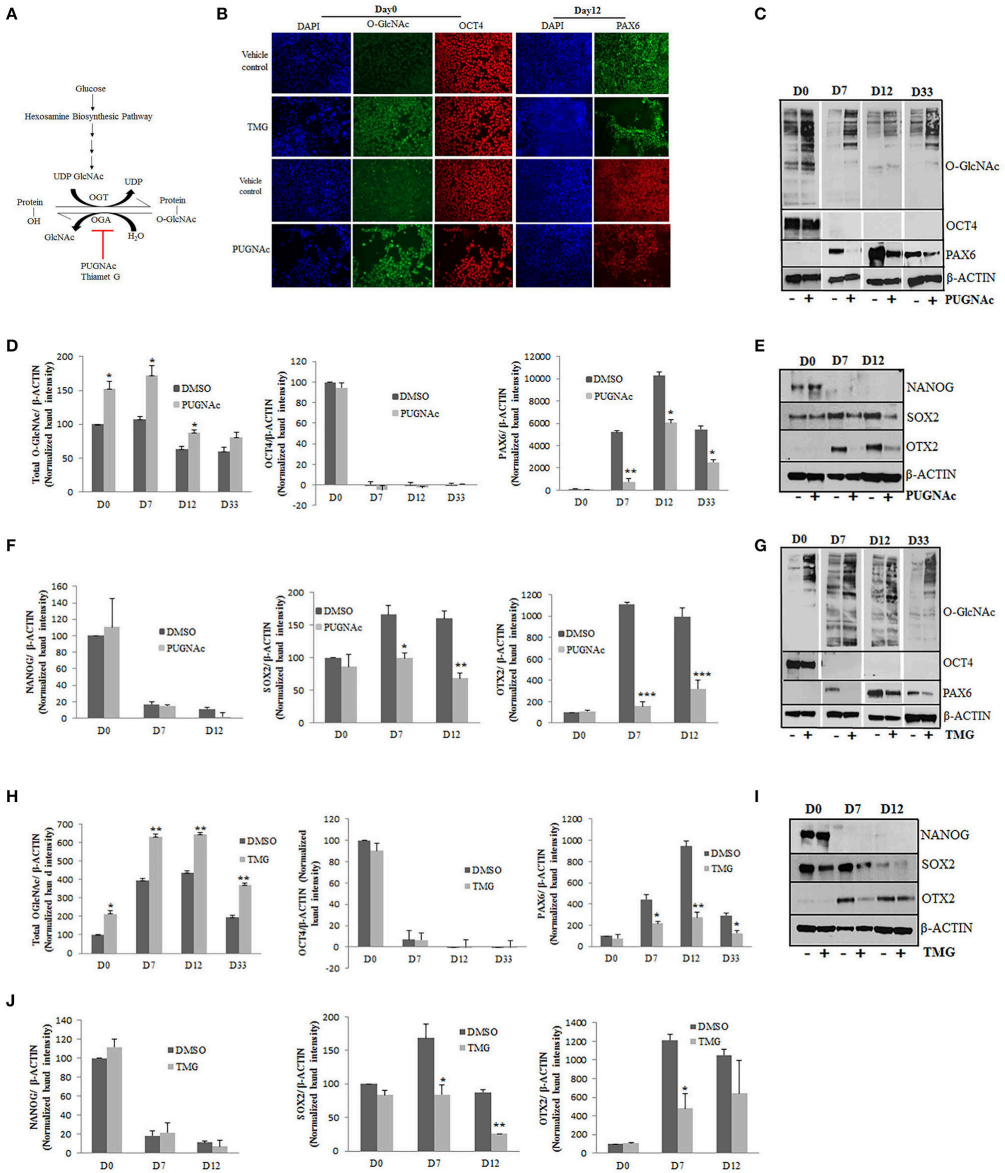
Effects of higher O-GlcNAc levels on the expression of pluripotency and neural progenitor markers. **(A)** The total levels of O-GlcNAcylation on target proteins could be elevated by increased glucose levels or by using specific inhibitors for enzyme OGA. H9 cells were treated with O-GlcNAcase inhibitors, PUGNAc (100 μM) and TMG (40 μM) for 3 days before differentiation into neural progenitors using dual SMAD inhibition protocol. **(B)** Immunostaining of PUGNAc, TMG treated and vehicle control cells from Day0 of differentiation using anti O-GlcNAc (RL2) and OCT4 antibodies and at Day12 of differentiation for Pax6 expression. DAPI represents nuclear staining. Scale bar = 100 μm. **(C,G)** Western blot analysis of PUGNAc and TMG treated as well as vehicle control samples was performed from D0, D7, D12, and D33 of cortical differentiation of H9 cells for O-GlcNAc, OCT4 and PAX6. The expression of β-*ACTIN* was used as loading control. **(D,H)** Densitometric quantitation of western blots from panel **(B,F)**. The values are expressed as percentage, relative to the values for D0-DMSO samples set as 100%. The data are represented as mean ± standard deviation. ^*^p ≤ 0.05, ^**^p ≤ 0.01, ^***^p ≤ 0.001 (two-tailed unpaired Student's *t*-test). **(E,I)** Western blot analysis of PUGNAc, TMG and vehicle treated control samples at D0, D7, and D12 of neural induction for NANOG, SOX2, and OTX2 expression. The expression of β-*ACTIN* was used as loading control. **(F,J)** Densitometric quantitation of western blots from panel **(D,H)**. The values are expressed as percentage, relative to the values for D0-DMSO samples set as 100%. The data are represented as mean ± standard deviation. ^*^p ≤ 0.05, ^**^p ≤ 0.01, ^***^p ≤ 0.001 (two-tailed unpaired Student's *t*-test). The data are representative of three independent biological replicates.

Increased levels of glucose inside the cells will fuel some of the glucose (3–5%) toward hexosamine biosynthesis pathway (HBP) resulting in increased production of end product, UDP-GlcNAc. Increased UDP-GlcNAc levels which is the substrate of enzyme OGT will result in increased O-GlcNAcylation of nucleocytoplasmic proteins. Inhibition of enzyme OGA which is responsible for removal of O-GlcNAc moiety from proteins could also lead to increased O-GlcNAcylation, mimicking glucose induced higher O-GlcNAcylation. Several small molecule inhibitors of OGA are being utilized as tools to understand the role of protein O-GlcNAcylation inside the cells (Figure 2A) (Kim et al., 2014; Trapannone et al., 2016). Therefore, to understand the effect of chronically elevated O-GlcNAc levels on the differentiation of hESCs toward neural fate, we employed two commonly used inhibitors of enzyme OGA, PUGNAc and Thiamet G (TMG). PUGNAc is a potent inhibitor of OGA and has been used in several key studies which addressed the role of O-GlcNAc modification on different cellular functions (Vosseller et al., 2002; Arias et al., 2004). However, recent studies have suggested off target effects of PUGNAc mainly due to its effect on inhibition of other β-hexosaminidases (Mehdy et al., 2012). We therefore used another potent inhibitor of OGA, ThiametG or TMG along with PUGNAc which has been shown to be highly selective for OGA in comparison to other hexosaminidases in the brain (Yuzwa et al., 2008) therefore leaving lower possibility for off target effects.

As mentioned above, we cultured H9 cells in feeder free conditions on matrigel coated plates and treated them with PUGNAc (100 uM) or TMG (40 μM) at least 3 days before starting neural induction and tested the levels of total O-GlcNAc inside the cells by immunocytochemistry using specific antibody against this modification. Undifferentiated H9 cells showed increased levels of O-GlcNAc when treated with TMG or PUGNAc in comparison to vehicle treated (DMSO) control cells (Figure 2B). Having confirmed the elevated O-GlcNAc levels in the treated cells, we next tested the effect of this increase on the pluripotency using the marker OCT4 in these cells. No difference was observed in the expression of OCT4 in the treated cells compared to the vehicle treated control cells (Figure 2B). Next we tested the effect of elevated O-GlcNAc levels on the expression of neural stem cell specific maker PAX6 on day 12 of neural induction. We observed a reduction in the number of PAX6 positive cells in both TMG and PUGNAc treated cultures in comparison to vehicle control culture. In order to validate these results quantitatively, we performed western blotting analysis for total O-GlcNAc, OCT4, and PAX6 using protein samples prepared from the TMG and PUGNAc treated cultures on day0 (undifferentiated), day7, day12, and day33 (neural induction) and compared them to vehicle treated control cells. As seen with immunocytochemistry, the cells treated with TMG and PUGNAc showed increased levels of O-GlcNAc modification (Figures 2C,D,G,H). Similarly, while the levels of OCT4 did not change in the TMG and PUGNAc treated samples compared to the vehicle control, it reduced significantly in both samples from day7 of neural differentiation as expected. These results suggested that increased O-GlcNAc modification had no effect on neural induction. On the other hand the expression of neuronal stem cell specific marker, PAX6 was reduced in both TMG and PUGNAc treated samples compared to vehicle control at all three stages of neural differentiation analyzed. In contrast to this, in the vehicle treated control cells, as expected, the levels of PAX6 gradually increased from day0 to day12 of neural induction, peaked at day12 and gradually decreased by day33 of differentiation (Figures 2C,D,G,H). In an effort to investigate this further, we verified whether these effects are also seen with other markers of pluripotency and neural progenitor cells. We therefore analyzed the expression of NANOG (pluripotency marker), SOX2 (which expresses both in ESCs as well as neural progenitor cells) (Zhou et al., 2016) and OTX2, (anterior telencephalic transcription factor) (Acampora et al., 1999) from day0, day7, and day12 of neural induction using PUGNAc, TMG and vehicle treated control cells. Our results showed no difference in the expression of NANOG between PUGNAc, TMG and vehicle treated control cells at day0 while as expected the expression of NANOG was down regulated on day7 onwards in both sets. The expression of SOX2 which expresses both in ESCs as well as neural progenitor cells was reduced in both TMG and PUGNAc treated cells only after neural induction. Similarly, as noted for PAX6, the expression of OTX2 was significantly reduced in both PUGNAc and TMG treated cells from day7 onwards. The expression of β-*ACTIN* which was used as control remained unchanged at all three time points of differentiation between PUGNAc, TMG and vehicle control cells (Figures 2E,F,I,J). Taken together, these results suggested that the expression of pluripotency markers, OCT4 and NANOG did not change by increased O-GlcNAc levels while the expression of neural progenitor markers, PAX6 and OTX2 were reduced due to higher O-GlcNAc levels. The expression of SOX2 which expresses both in ESCs and neural progenitors was reduced only after neural induction due to increased O-GlcNAc modification levels.

### Effect of higher O-GlcNAc levels on the transcription of neural stem cell specific genes

**Figure 3:**
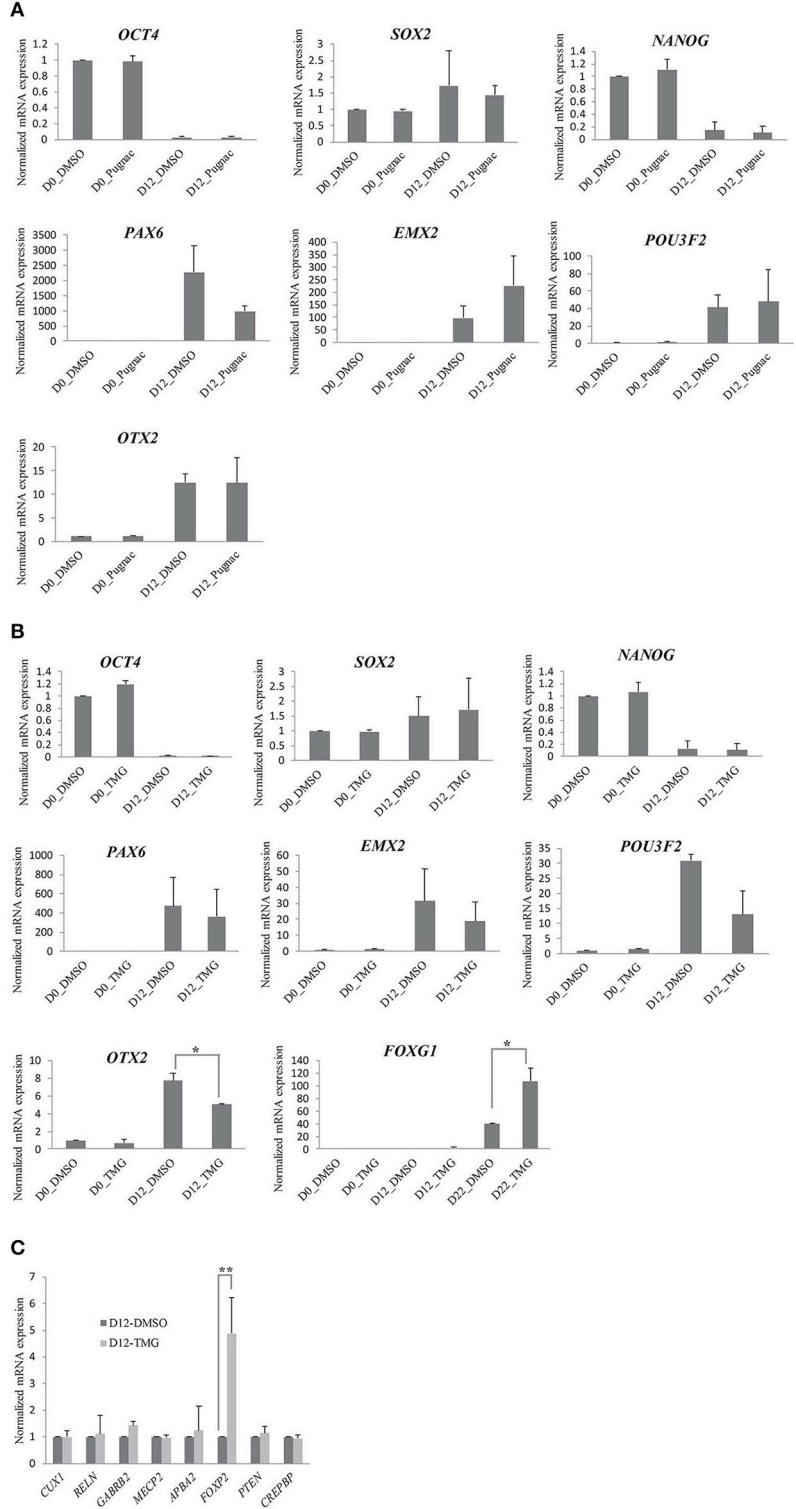
Higher O-GlcNAc levels selectively affect the transcription of neural progenitor specific genes. H9 cells were treated with O-GlcNAcase inhibitors, PUGNAc (100 μM) and TMG (40 μM) for 3 days before differentiation into neural progenitors using dual SMAD inhibition protocol. **(A,B)** PUGNAc, TMG treated and vehicle control cells were collected from Day0 and Day12 of neural induction and analyzed for mRNA expression by qRT-PCR for the indicated genes. **(C)** TMG treated and vehicle control cells were collected at Day12 of neural induction and analyzed for mRNA expression by qRT-PCR for the indicated genes using gene specific primers. In **(A–C)**, the expression of β*-ACTIN* gene was used to normalize the expression. The bars represent normalized fold mRNA expression with values of vehicle control samples on Day0 set as 1.The data are represented as mean ± standard deviation. ^*^p ≤ 0.05, ^**^p ≤ 0.01 (two-tailed unpaired Student's *t*-test). The data are representative of three independent biological replicates.

Having seen that the increase in O-GlcNAc modification alters neural progenitor differentiation, we next looked for the mechanisms by which this is mediated. Protein O-GlcNAcylation has been associated with gene expression changes at the transcriptional level by various mechanisms such as O-GlcNAcylation of specific transcription factors, histone modification on the promoter DNA or O-GlcNAcylation of RNA Pol II CTD (Ozcan et al., 2010; Fujiki et al., 2011; Ranuncolo et al., 2012). To identify the effects of higher O-GlcNAc on transcription, we first sought to determine the effect on the transcription of genes important for the maintenance of pluripotent and neural stem cell fate. As discussed above, increased levels of O-GlcNAc did not show any change in the protein levels of OCT4 and NANOG while the expression of neural progenitor markers, PAX6, OTX2, and SOX2 were reduced (Figure 2). We further verified this by qRT-PCR using samples prepared from vehicle control, TMG and PUGNAc treated cells from day0 and day12 of differentiation. As was observed for protein, the RNA levels of *OCT4* and *NANOG* did not change upon increased O-GlcNAc modification (Figures 3A,B). The RNA levels of *SOX2* and *PAX6* also remained unchanged in contrast to what was seen with the protein expression. The expression of the other neuronal progenitor *OTX2* did not change in the PUGNAc treated samples while the TMG treated samples showed a significant reduction of *OTX2* expression (*p* < 0.05) (Figures 3A,B) which correlates with the reduction seen at protein levels by western blotting (Figures 2I,J). We have also tested the expression of *EMX2* (a dorsal telencephalic marker) (Cecchi, 2002) and *POU3F2* (a cortical progenitor and neuronal marker) (Dominguez et al., 2013) and did not find any significant differences in the RNA levels in both PUGNAc and TMG treated samples compared to that of the vehicle control. In these experiments we used two inhibitors of OGA, PUGNAc and TMG and compared the results obtained due to increased O-GlcNAcylation. Since most of the results obtained as discussed above (Figures 2, 3A,B) are similar for both PUGNAc and TMG, we decided to focus on inhibition by TMG for rest of the experiments because of its higher specificity for OGA inhibition (Yuzwa et al., 2008).

We next tested the RNA expression of *FOXG1* which is an important transcriptional regulator of neural stem cell fate and is needed for neural stem cell self-renewal and neurogenesis (Martynoga et al., 2005). Overexpression of *FOXG1* has been recently shown to be responsible for idiopathic autism spectrum disorder (ASD) in an induced pluripotent stem cell (iPSC) model of the disease (Brunetti-Pierri et al., 2011). We found that during neural induction of H9 cells, *FOXG1* expression started by day12 which increased almost forty fold by day22 when compared to undifferentiated cells (day0). We therefore tested the expression of *FOXG1* at day0, day12, and day22 of differentiation from the vehicle control and TMG treated samples. We found that the expression of *FOXG1* was significantly increased in TMG treated samples on day22. We further analyzed the expression of several other genes (*CUX1, RELN, GABRB2, MECP2, APBA2, FOXP2, CREBBP*, and *PTEN*) which are expressed in neural progenitor cells and are shown to be associated with defective neurogenesis or neurodevelopmental abnormalities (van Loo and Martens, 2007; van de Leemput et al., 2014). None of these genes tested here showed any change in the RNA expression except *FOXP2* which increased by almost 5-fold (*p* < 0.05) in TMG treated cells compared to that of vehicle control (Figure 3C). Taken together these results suggested that there was no significant defect in the expression of *PAX6* gene at the RNA level in higher O-GlcNAc modification as opposite to its effect seen at the protein level. The expression of *OTX2* was reduced both at RNA and protein levels in TMG treated cells suggesting that higher O-GlcNAc levels may affect gene expression at transcriptional level (*OTX2*) or at post-transcriptional levels (*PAX6*). The other markers or effectors of neural progenitors did not show any change in RNA expression except for *FOXG1* and *FOXP2*, both of which were upregulated, suggesting further that the effects of higher O-GlcNAc on transcription are gene specific.

### Increase in O-GlcNAc levels result in premature neurogenesis of hESCs during cortical differentiation

**Figure 4:**
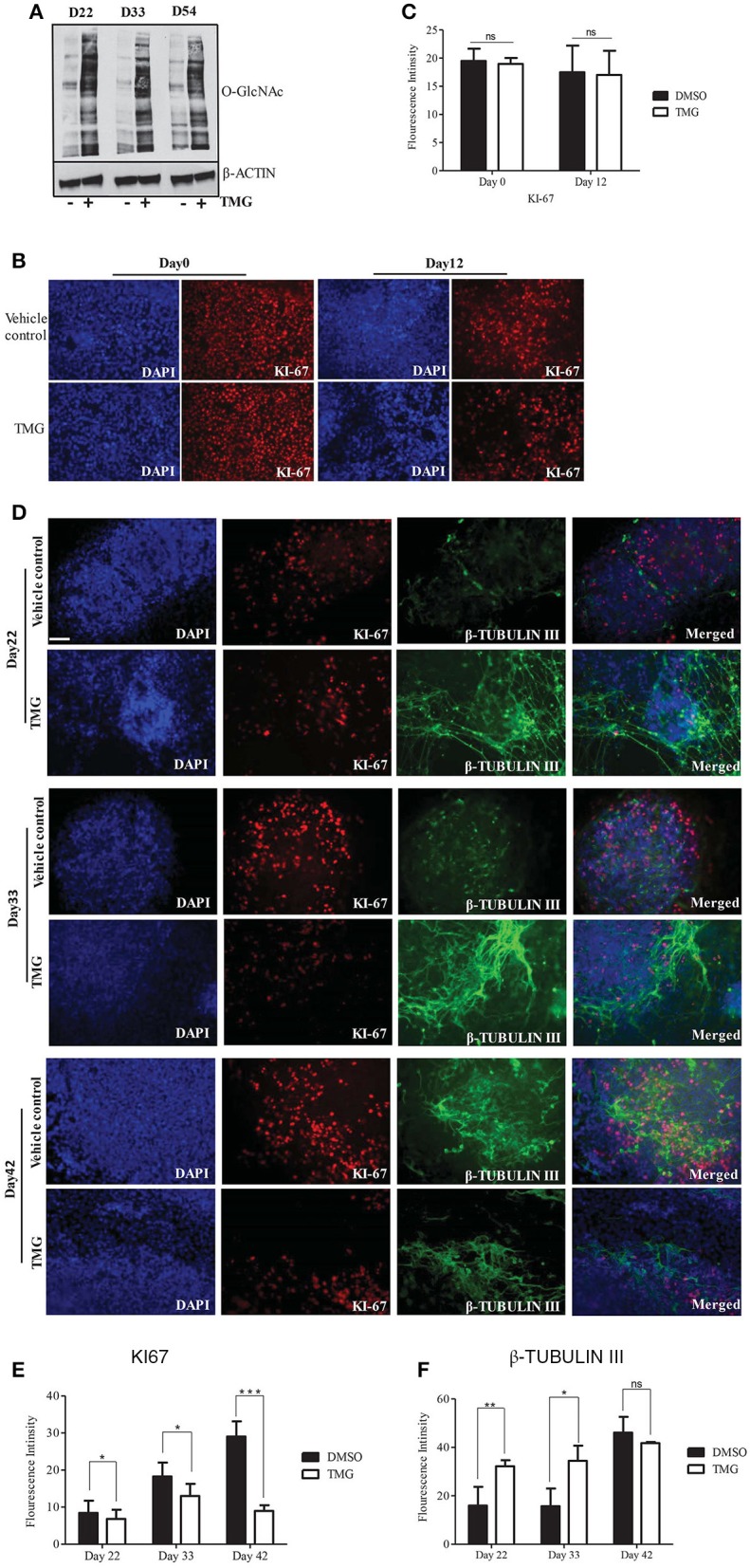
Higher O-GlcNAc levels affect neural progenitor proliferation and differentiation during corticogenesis. Neural progenitor cells from Day12 of neural induction were differentiated for cortical neurons in N2/B27 media for up to day 54. **(A)** Western blot analysis of TMG treated and vehicle control samples were performed from D22, D33, and D54 of cortical differentiation of neural progenitors to detect total O-GlcNAc levels. The expression of β-*ACTIN* was used as loading control. The data are representative of three independent biological replicates. **(B)** Immunostaining of TMG treated and vehicle control H9 cells for KI-67 at Day0 and Day12 of neural induction. **(C)** The fluorescence intensity of images from panel **(B)** was quantified using ImageJ (Fiji) software. The data are represented as mean ± standard deviation. **(D)** Immunostaining of cortical differentiated TMG treated and vehicle control cells for KI-67 and β-Tubulin III at Day22, Day33, and Day42 of differentiation. In **(B,C)**, the cells were imaged with fluorescence microscope. DAPI represents nuclear staining. Scale bar = 100 μm.The data are representative of three independent biological replicates. **(E,F)** The fluorescence intensity of images from panel **(D)** was quantified using ImageJ (Fiji) software. The data are represented as mean ± standard deviation. ^*^p ≤ 0.05, ^**^p ≤ 0.01, ^***^p ≤ 0.001 (two-tailed unpaired Student's *t*-test).

The genes *PAX6, OTX2, SOX2, FOXG1*, and *FOXP2*, whose expressions were altered by higher O-GlcNAc levels are important regulators of neural stem cell fate of dorsal telencephalon, suggesting for the possibility that this could affect downstream cortical neurogenesis. In order to verify this, we differentiated neural progenitor cells obtained on day12 of neural induction toward cortical neurogenesis as described above. The cells from day12 of neural induction were cultured in N2/B27 media for 8–10 weeks of neural induction with TMG to keep the levels of O-GlcNAc chronically elevated, while the other set was treated with vehicle control. We collected the cells from different stages of cortical differentiation i.e., day22, day33, and day54 to confirm whether the levels of total O-GlcNAc were higher throughout the course of cortical differentiation. Western blots performed with protein samples from these three time points showed increased O-GlcNAc levels compared to the vehicle control (Figure 4A). We then tested the effect of higher O-GlcNAc levels on cell proliferation by immunostaining using cell proliferation marker, KI67. We found that the numbers of KI-67 positive cells were similar in the vehicle control and TMG treated cells at day0 and day12 of differentiation (Figures 4B,C). Under normal differentiation, the early born deep layer neurons start appearing by day35 of neural induction which can be detected by immunostaining of neuron specific β-TUBULIN III gene (van de Leemput et al., 2014). However, we observed that in TMG treated cells, neurons started to appear prematurely by day22. We verified this further by immunostaining using the cell proliferation marker KI-67 and neuronal specific marker β- TUBULIN III on day22, day33, and day42 of neural differentiation. The staining for the KI-67 showed more KI-67 positive cells in vehicle control in comparison to TMG treated cells at all three time points (Figures 4D,E). On the other hand, cells treated with TMG showed positive staining for β-TUBULIN III on day22 whereas the vehicle control treated cells did not show any positive β-TUBULIN III expression (Figure 4D). Further, the expression of β-TUBULIN III remained high on day33 and day42 in the TMG treated cells whereas in the vehicle control cells, its expression increased gradually from day22 to day42. On day42 both TMG and the vehicle treated control cells showed similar numbers of β-TUBULIN III positive neurons (Figures 4D,F). Together these results suggested that the increase in the O-GlcNAc levels were associated with premature neurogenesis and decrease cell proliferation during the course of cortical neurogenesis of hESCs although this has no effect on the proliferation of undifferentiated (day0) and neural progenitor cells (day12).

### Higher O-GlcNAc levels are associated with aberrant akt phosphorylation and cellular apoptosis during cortical neurogenesis

**Figure 5:**
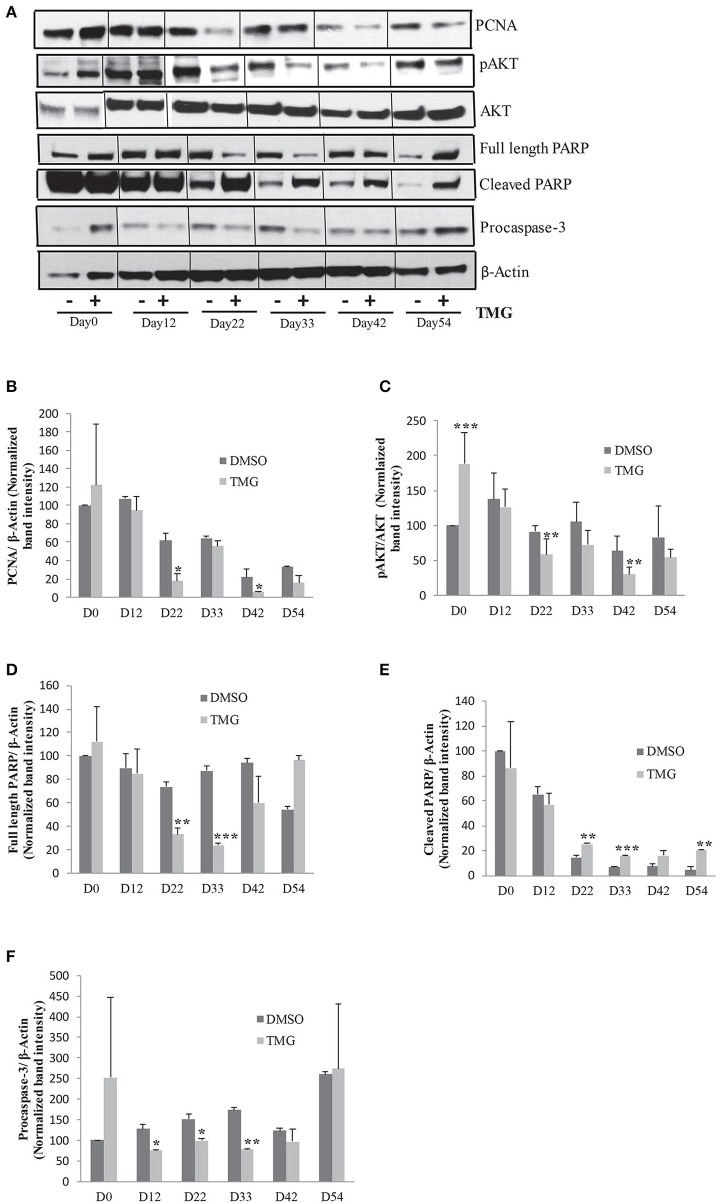
Higher O-GlcNAc levels cause cellular apoptosis. **(A)** Western blot analysis of TMG treated and vehicle control samples from Day0 to Day54 of cortical differentiation to detect the expression of PCNA, p-AKT308, AKT1,2,3, PARP (full length and cleaved), and Caspase-3. The expression of β-*ACTIN* was used as loading control. **(B–F)** Densitometric quantitation of western blots from panel **(A)**. The values are expressed as percentage, relative to the values for D0-DMSO samples set as 100%. The data are represented as mean ± standard deviation. ^*^p ≤ 0.05, ^**^p ≤ 0.01, ^***^p ≤ 0.001 (two-tailed unpaired Student's *t*-test). The data are representative of three independent biological replicates.

Higher O-GlcNAc levels have been shown to be associated with cellular apoptosis in different cell and tissue types including heart muscle, pancreatic beta cells and neurons (Liu et al., 2000, 2004; Dassanayaka and Jones, 2014; Zhu et al., 2015; Ning et al., 2017). We therefore reasoned that the reduction in the numbers of KI-67 positive cells observed in TMG treated samples during cortical differentiation (Figures 4D,E) could be a result of increased cell death. To test this, we analyzed the level of apoptosis by performing western blotting using antibodies against full length and cleaved PARP as well as pro and cleaved CASPASE-3. We found that the level of cleaved PARP was increased in TMG treated samples during cortical differentiation stages, day22, day33, day42, and day54. However, samples from day0 and day12 did not show any elevation in the levels of cleaved PARP compared to the vehicle treated control (Figures 5A,D,E). Although we were not able to detect cleaved CASPASE-3 in these samples, the levels of pro CASPASE-3 itself was decreased significantly from day12 to day33 in TMG treated samples, suggesting that activation of caspase cascade could be one mechanism leading to apoptosis (Figures 5A,F). We further confirmed the effect of TMG on cell proliferation by checking the expression of another cell proliferation marker, PCNA by western blotting. The levels of PCNA were reduced in TMG treated samples at all of the stages of cortical differentiation, but significantly on days 22 and day42 (Figures 5A,B). No difference in the levels of PCNA was noted from day0 and day12 samples. These results together suggest that increasing the levels of O-GlcNAc result in reduced cell proliferation and increased apoptosis during cortical neurogenesis. However it has no effect on undifferentiated hESCs or early stages of neural differentiation (day12).

Previous studies have suggested that one mechanism by which increased O-GlcNAc levels lead to cellular apoptosis is due to its effect on AKT signaling, a key signaling pathway needed for cell proliferation and cell survival (Shi et al., 2015). We tested this possibility by performing western blotting using samples from all of the stages of neuronal differentiation as before with antibodies specific for Thr308 p-AKT and total AKT1,2,3. We found while there was no difference in the levels of total AKT, the levels of p-AKT was reduced in TMG treated samples from day22 of differentiation onwards although this reduction was statistically significant only on day22 and day42. Interestingly, the level of p-AKT was significantly high in TMG treated samples on day0 of differentiation (Figures 5A,C). The expression of β-*ACTIN* was used as loading control.

### Increased levels of O-GlcNAc result in transcriptional defects during cortical neurogenesis

**Figure 6:**
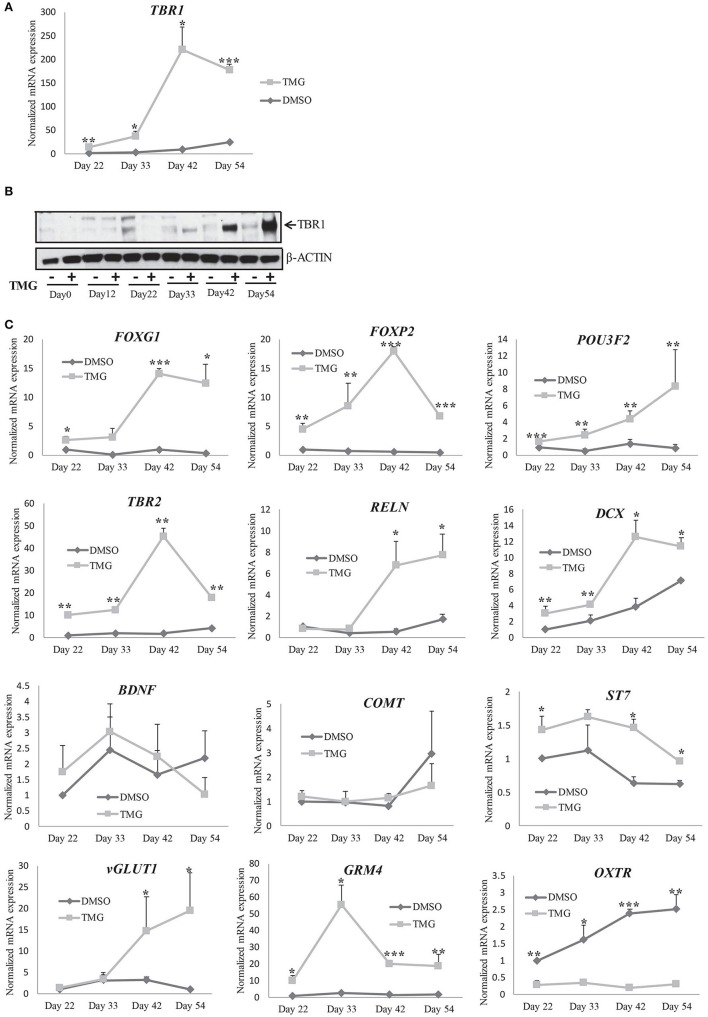
Gene expression changes during cortical differentiation in the presence of higher O-GlcNAc. **(A)** mRNA expression analysis of *TBR1* gene by qRT-PCR in TMG treated and vehicle control samples from indicated time points (Day33 to Day54) of cortical differentiation using gene specific primers. The expression of β*-ACTIN* gene was used to normalize the expression. The bars represent normalized fold mRNA expression with values of vehicle control samples at Day22 set as 1. The data are represented as mean ± standard deviation. ^*^p ≤ 0.05, ^**^p ≤ 0.01, ^***^p ≤ 0.001 (two-tailed unpaired Student's *t*-test). The data are representative of three independent biological replicates. **(B)** Western blot analysis of TMG treated and vehicle control samples were performed at the indicated time points (Day0 to Day54) of cortical differentiation to detect the expression of TBR1. The expression of β-*ACTIN* was used as loading control. **(C)** mRNA expression analysis of the indicated genes by qRT-PCR using TMG treated and vehicle control samples from the indicated time points (Day33 to Day54) of cortical differentiation using. The expression of β*-ACTIN* gene was used to normalize the expression. The bars represent normalized fold mRNA expression with values of vehicle control samples at Day22 set as 1. The data are represented as mean ± standard deviation. ^*^p ≤ 0.05, ^**^p ≤ 0.01, ^***^p ≤ 0.001 (two-tailed unpaired Student's *t*-tst). The data are representative of three independent biological replicates.

Over the years, different genes have been shown to be involved in the regulation of cell proliferation and differentiation during cortical neurogenesis and defects in their expression have been shown to be associated with different neurodevelopmental disorders (Chen et al., 2014; Gigek et al., 2015; Maussion et al., 2015). T-box brain 1 (*TBR1*) is a transcription factor expressed at a high level in early born deep layer cortical neurons and was shown to be required for differentiation of cortical progenitor cells (Hevner et al., 2001; Bedogni et al., 2010). We analyzed the expression of *TBR1* at RNA level by qRT-PCR in the TMG treated samples and compared them to vehicle treated control cells from different stages of differentiation after neural induction (day22, day33, day42, and day54). The expression of *TBR1* increased steadily in the vehicle control cells by almost 25-fold on day54 compared to day22. However the expression in TMG treated samples was about 13-fold up on day22 of differentiation compared to the vehicle treated control cells which further increased by 25-fold by day42 of differentiation (Figure 6A). We then checked whether differences in the expression of *TBR1* observed at RNA levels was also seen at the protein levels. Western blotting was performed for TBR1 from TMG and vehicle control cells at all four stages of differentiation as for RNA analysis. We found that the expression was significantly increased at all stages in TMG treated cells compared to vehicle treated control cells as observed with RNA expression (Figures 6A,B). These results together suggested that the increased expression of *TBR1* in TMG treated cells could have influenced neuronal differentiation resulting in premature neurogenesis observed (Figures 4D,F). We further analyzed a number of transcription factors, *FOXG1, FOXP2*, and *POU3F2* (*BRN2*) which also influence cortical neurogenesis and are known to be involved in various neurodevelopmental disorders (van de Leemput et al., 2014). The RNA expression of these genes was analyzed by qRT-PCR using TMG treated samples and vehicle treated control. Expression of these genes at day12 of neural induction (Figure 3A) showed that both *FOXG1* and *FOXP2* were significantly up-regulated in TMG treated samples while the expression of *POU3F2* did not show any significant change. Since these genes continue to express during the course of cortical neurogenesis and are known to regulate progenitor cell differentiation, we checked their expression from later stages of differentiation (day22, day 33, day42, and day54). We found that increased expression of *FOXG1* and *FOXP2* in TMG treated samples continued from day12 of differentiation, whereas, *POU3F2* which did not show any change in expression on day12 of differentiation was up-regulated from day33 (Figure 6C). Interestingly, we also found that the expression of *TBR2* (*EOMES*) was significantly up-regulated in TMG treated samples at all stages of cortical differentiation (Figure 6C). During cortical neurogenesis, *TBR2* is specifically expressed in the intermediate progenitor cells formed after differentiation of PAX6 positive radial glia cells (Englund et al., 2005). Some of these TBR2 positive cells then differentiate into postmitotic neurons positive for TBR1. An increased level of *TBR2* in TMG treated samples may therefore suggest an increase in the number of intermediate progenitor cells which could then result in increased number of differentiated neurons as observed in our analysis (Figures 4D,F). We also checked the expression of Reelin (*RELN*) and doublecortin (*DCX*) both of which are expressed in the developing cerebral cortex. We did not see any change in the expression of *RELN* in neural progenitors (Figure 3C). When checked during cortical differentiation, *RELN* did not show any change in expression up to day33 which then increased from day42 onwards in the TMG treated samples (Figure 6C).The expression of *DCX* was high in TMG treated samples from day22 with the highest up-regulation seen on day42 (Figure 6C). The expression of other genes, *BDNF, COMT*, and *ST7* also tested during cortical differentiation period, day22 onwards showed less than 2 fold changes during the course of differentiation, although the changes for *ST7* were found to be statistically significant (*p* < 0.05) (Figure 6C). We also tested the expression of the vesicular glutamate transporter (*vGLUT1*) which is expressed in glutamatergic neurons of the cortex as majority of neurons differentiated using this protocol are presumably glumatergic projection neurons (Shi et al., 2012a; van de Leemput et al., 2014). Interestingly, no difference in the expression of *vGLUT1* was noted between the vehicle treated control cells and TMG treated samples on day22 and day33 of differentiation, however expression was significantly up-regulated on day42 and day54 of differentiation on the TMG treated cells (Figure 6C). Another glutamate neurotransmitter receptor, *GRM4* also showed significantly increased expression at all stages of cortical neurogenesis. In contrast to this, the expression of oxytocin receptor (*OXTR*) was significantly down regulated in TMG treated samples at all stages of corticogenesis (Figure 6C).

Together our results suggested that elevated levels of O-GlcNAc modification result in increased RNA expression of different transcription factors which are either involved in neuronal differentiation or express in the post-mitotic neurons which in turn might have a role in premature neurogenesis observed (Figures 4D,F). We could not however differentiate whether increased expression of these genes is due to a direct effect of higher O-GlcNAc modification on the transcriptional machinery resulting in increased transcription or due to the presence of higher number of differentiating and lower numbers of proliferating cells in the presence of higher O-GlcNAc levels. Further investigation in this direction may clarify the exact role of O-GlcNAc modification on transcription during the process of developmental neurogenesis.

## Discussion

Epidemiological as well as studies using animal models have established a link between the maternal nutritional status and embryonic neurodevelopment. Over or under nutrition during pregnancy has been shown to cause adverse neurodevelopmental outcomes in the offspring. Further, metabolic syndrome which includes type 2 diabetes, hypertension, cardiovascular diseases and obesity which is linked to the maternal nutritional status has been associated with various neurodevelopmental disorders (Ornoy et al., 2001, 2015; Kinney et al., 2003; Krakowiak et al., 2012; Lopaczynski, 2012; Xu et al., 2014a; Hami et al., 2015). One critical change which occurs as a result of altered metabolism is hyperglycemia and this increases the levels of O-GlcNAcylation. So, in this study we have verified the effect of higher O-GlcNAc levels on human embryonic neurogenesis by directed differentiation of hESCs into neurons of cortical fate. This has been shown to recapitulate the early events of human embryonic corticogenesis by spatial and temporal expression of stage specific markers and is highly cell autonomous (Shi et al., 2012a; van de Leemput et al., 2014). Further, whole genome transcriptomic analysis of these cells at different stages of differentiation suggested that the cortical neurons generated are closely associated with pre-frontal cortex (PFC) region of the brain (van de Leemput et al., 2014). This is of significance as the PFC is associated with various neurodevelopmental disorders such as Autism spectrum disorder (ASD), Attention deficit hyperactivity disorder (ADHD), Intellectual disability (ID) and schizophrenia (Rubenstein, 2011; Schubert et al., 2015). Our results suggest that, higher levels of O-GlcNAc did not affect pluripotency as there was no change in the expression of pluripotency markers, OCT4 and NANOG. However, we noticed the expression of the other pluripotency marker SOX2, which is also a key regulator of neural induction as well as differentiation was significantly reduced at protein level after neural induction (Zhou et al., 2016). Earlier studies have reported that both the transcription factors, OCT4 and SOX2 are O-GlcNAcylated at specific amino acid residues and changes in this modification affects the expression of their downstream target genes (Jang et al., 2012). We found that in normal conditions, the levels of total O-GlcNAc are higher in undifferentiated H9 cells in comparison to all other stages of neural differentiation (Figure S2). Similar studies done on hESCs as well as in animal models have also reported that the levels of total O-GlcNAc reduce as the cells start to differentiate (Liu et al., 2012; Maury et al., 2013). This suggests that increasing the levels of O-GlcNAc further by PUGNAc or TMG in the undifferentiated cells may not affect pluripotency however reduced expression of SOX2due to high O-GlcNAc levels may affect neural differentiation (Zhao et al., 2004). SOX2 suppresses the differentiation of hESCs into non-neural lineages by its interaction with histone variant H2A.Z and recruitment of polycomb repressor complex 2 (PRC2) to specific developmental genes (Zhou et al., 2016). Therefore reduced numbers of neuroepithelial marker PAX6 expressing cells observed due to both PUGNAc and TMG treatment in this study could be as a result of decreased expression levels of SOX2. In the developing CNS, SOX2 is known to act cooperatively with PAX6 to regulate neural stem cell proliferation and differentiation (Gómez-López et al., 2011). Earlier studies have shown that the reduced expression of SOX2 in neural progenitor cells hinders their self-renewal and proliferation, promoting their earlier exit from cell cycle and premature terminal differentiation (Graham et al., 2003). Similarly, PAX6 is a highly conserved transcription factor, expressed in the developing cortex and is essential for normal cortical neurogenesis (Georgala et al., 2011) as it maintains the balance of neural stem cells' self-renewal and differentiation (Sansom et al., 2009). Both loss and gain of function mutations of Pax6 showed reduced progenitor cell proliferation and an increase in early neurogenesis (Warren et al., 1999; Quinn et al., 2007), a feature we noticed in the TMG treated samples. We also noticed that the expression of another prominent neural progenitor marker and transcription factor, OTX2 was also reduced in TMG treated samples. Earlier studies have suggested that OTX2 is required for cortical neurogenesis and in patterning the developing brain through a gene-dosage dependent mechanism (Simeone, 1998; Acampora et al., 1999). Further, our studies have also identified that the expression of two other genes important for cortical differentiation of the neural progenitor cells, FOXG1 and FOXP2 are significantly upregulated in TMG treated samples. FOXG1 is a transcription factor needed for progenitor proliferation and cortical specification (Martynoga et al., 2005). Interestingly, duplications of FOXG1 on chromosome 14q12 has been shown to be associated with epilepsy, mental retardation, and speech impairment (Brunetti-Pierri et al., 2011). Similarly, FOXP2 is also shown to regulate early cortical neurogenesis and a defect in its expression is associated with speech impairment (Enard et al., 2002; Tsui et al., 2013; Chiu et al., 2014). It is well known that during cortical neurogenesis, some of the PAX6 expressing neural progenitor or radial glia cells at the ventricular surface differentiate into intermediate progenitor cells (IPCs) expressing the transcription factorTBR2 at the subventricular zone (Kowalczyk et al., 2009). These IPCs divide asymmetrically giving rise to a progenitor cell and a post mitotic pyramidal neuron expressing TBR1 (Pontious et al., 2008). This gives rise to a sequential expression of transcription factors, PAX6 (radial glia cell), TBR2 (IPCs) and TBR1 (post mitotic neurons) in the developing cortex of rodent brain although variations to this rule have been noted in human (Englund et al., 2005). We have seen that the presence of high O-GlcNAc levels lead to reduced expression of PAX6, reduced cell proliferation, increased neurogenesis and increased levels of TBR2 and TBR1 expression at early stages of cortical differentiation. We also found the presence of many other markers of differentiated cortical neurons such as *DCX, vGLUT1*, and *GRM4* being significantly up-regulated as a result of high O-GlcNAc levels. In contract, mRNA expression of oxytocin receptor (*OXTR*) was significantly reduced in the presence of high O-GlcNAc modification. Oxytocin is known to play critical roles in social and emotional behavior and a decrease in the expression of its receptor, OXTR is shown to be associated with social anxiety (Puglia et al., 2015; Cappi et al., 2016), a feature commonly observed in many neurodevelopmental disorders.

Defects in cell proliferation and differentiation are common features of various neurodevelopmental disorders (Gigek et al., 2015; Ernst, 2016). Based on our results, it could be speculated that increased O-GlcNAcylation could result in defects in various signaling pathways due to its effect on the expression of various modulators of neurogenesis. The phenotypes that we observed here due to increased O-GlcNAc levels such as reduced progenitor proliferation and premature neurogenesis as well as apoptosis could therefore result from one or many different mechanisms.

Increased apoptosis due to the presence of high O-GlcNAc levels have been reported in various cell types including pancreatic β cells, cardiac myocytes and neurons (Liu et al., 2000, 2004; Dassanayaka and Jones, 2014). These studies also have implicated that AKT signaling pathway which is involved in cell survival by inhibition of cellular apoptosis (Shi et al., 2015) has been altered by high O-GlcNAc levels. We found that the phosphorylation of AKT was indeed reduced in TMG treated samples at almost all stages of cortical differentiation from day22 onwards. Further, the expression of the apoptotic marker, cleaved PARP increased, as the levels of pAKT reduced, at all stages of cortical differentiation. The question then arises about the mechanisms behind higher O-GlcNAc associated phenotypes observed in our study. One possible explanation is that, the reduced levels of p-Akt may lead to reduced cell survival and apoptotic cell death. However, the cells which escape apoptosis may end up differentiating into IPCs and then into neurons rather than self-renewal leading to reduced numbers of proliferating cells as observed here. The neurodevelopmental disorders have a complex etiology involving a large number of genes most of which are known to be required for proper neurogenesis (Rubenstein, 2011; Schubert et al., 2015). Identifying a single molecular mechanism or a signaling pathway such as HBP pathway or protein O-GlcNAcylation as identified here which seems to regulate the expression of several genes important for normal development and differentiation of neurons could help explore similar molecular mechanisms in models of neurodevelopmental disorders. Importantly this could be used for pharmacological intervention of several NDDs which probably have same etiology due to defects in the same genes during early stages of neurogenesis. Targeting HBP pathway or O-GlcNAc modification in case of hyperglycemia could be one strategy for early intervention during embryogenesis to avoid neurodevelopmental defects.

## Materials and methods

### Cell culture, differentiation and inhibitor treatment

We used commercially available hESC line, WA09 or H9 throughout this study which was purchased from WiCell Institute, Madison, WI, USA. The cells were cultured in feeder free condition on matrigel (Corning; Cat.#354227) coated tissue culture dishes in mTeSR1 medium (Stem Cell Technologies Cat.#05850) to maintain in pluripotent state. Previously published protocols (Chambers et al., 2009; van de Leemput et al., 2014) were adapted to induce neural differentiation of H9 cells in monolayer culture conditions. Briefly, 40,000–50,000 cells/cm^2^ were plated on a 24 well plate coated with matrigel and maintained in mTeSR1 medium until fully confluent. The medium was then replaced with neural induction medium comprising of 15% Knockout Serum Replacement (KO-SR Gibco; Cat. #10828028), 1% L-glutamine (100×-Gibco Cat.#25030081), 1%MEM (Hyclone; Cat.# SH40003.01), and 0.1% beta-mercaptoethanol (Gibco Cat.#31350010) in knockout DMEM (Gibco; Cat.#10829018) supplemented with LDN193189 (Stem Cell Technologies; Cat.#72142-1mg Lot #SCO4565), inhibitor BMP type 1 receptors (100 nM) and SB431542 (Milipore; Cat. #616461-5mg Lot#D00165595), activin receptor inhibitor (10uM) and N2 media [8.5mM glucose, 1x N-2 supplement (Gibco; Cat.#17502048) in DMEM/F12 (Gibco,Cat.#17330-032,1:1)]. To secure a dorsal telencephalic fate, the cultures were supplemented with sonic hedgehog antagonist, cyclopamine (Stem Cell Technologies; Cat#72072) at a final concentration of 1 uM from day3 of neural induction. The initial 5 days of differentiation included 100% of KSR media which was gradually replaced with N2 media from day 5 onwards as 25, 50, 75, and 100% N2. The differentiation was carried out for a total of 12 days after which the cultures were continued for cortical differentiation for another 8–10 weeks using the protocols published before (Chambers et al., 2009; van de Leemput et al., 2014) in N2/B27 (1:1) media with media change every alternate day. To increase total levels of O-GlcNac, cells were treated with O-GlcNAcase (OGA) inhibitors, O-(2-Acetamido-2-deoxy-D-glucopyranosylidene) amino N-phenyl carbamate, PUGNAc (3384-10MG, TOCRIS bioscience) and ThiametG, TMG (4390-10MG, TOCRIS bioscience) dissolved in DMSO according to manufacturer's instructions at a concentrations, 10mM and 40mM respectively. The working concentrations for PUGNAc (100 uM) and TMG (40 uM) were decided based on previous reports (Ostrowski and van Aalten, 2013). Same volume of DMSO alone was used as vehicle control in all of the treatments. The use of H9 cells was according to NIH guidelines (https://oir.nih.gov/sourcebook/ethical-conduct/special-research-considerations/use-human-stem-cells/guidelines-human-embryonic-induced-pluripotent-stem-cells), based on a letter of agreement signed between WiCell Institute (University of Wisconsin) and UAE University.

### Immunocytochemistry

For immunostaining experiments, cells were treated with PUGNAc or TMG and maintained and differentiated on 4-well plates (1.9 cm^2^, Nunc USA). On the day of staining, cells were washed with PBS and fixed with 4% formaldehyde for 10 min, permeabilized in PBS-2% triton-X for 10 min and blocked with 1% bovine serum albumin in PBS. After which the cells were incubated with primary antibodies at 4°C overnight. Alexa Fluor conjugated appropriate secondary antibodies were added to the cells for 1 h at room temperature, and after three washes with PBS, the cells were counterstained with nuclear stain DAPI [ThermoFisher Scientific DAPI (4′,6-Diadino-2-Phenylindole, Dihhydrochloride), Cat.#D1306]. Fluorescent images were taken with an inverted Axiovert 40 CFL fluorescence microscope (Carl Zeiss), equipped with AxioCam HRc (Carl Zeiss). The fluorescence intensity of images was quantified using ImageJ (Fiji) software.

### Protein extraction and quantitation

The cells, cultured in 24- (Corning, Cat.#3527) or 4-well plates (Thermo Scientific, Cat.# 176740) were washed with sterile 1x-D-PBS (Stem Cell Technologies, Cat.#37350) and gently scraped using a sterile cell scraper (Sarstedt, Cat.# 83.1832) in 1X RIPA buffer (Abcam, ab156034) containing 1X HALT Protease and 1X HALT Phosphatase Inhibitor Cocktail (Thermo Scientific, Cat.#1862209, Cat.#1862495). The crude lysate was centrifuged at 14,000 rpm at 4°C for 10 min. The supernatant containing the total cellular protein was quantitated with Pierce BCA Protein Assay Kit (Cat.#23225) using the Infinite M200Pro multimode microplate reader (Tecan). The quantitated protein was denatured by heating in NuPAGE LDS sample buffer (Life Technologies, NP0007) for 10 min at 70°C. 40 μg aliquots of the protein in sample buffer were frozen at −80°C until use.

### SDS-PAGE and western blotting

The concentration of protein for gel loading was determined based on the target being analyzed. Generally, 20–40 μg of protein was loaded along with the Precision Plus Protein Dual Color Standards (BioRad, Cat.#1610374) on Express Plus 4–12% PAGE gels (GenScript, Cat.# M41210 or M41215) and electrophoresed in 1X MOPS buffer (GenScript, M00138) at 100 V. Electroblotting was performed in 1X Towbin's buffer at a constant voltage of 100 V for 1 h at 4°C. After transfer, the PVDF membrane (Thermo Scientific, Cat.# 88518) was blocked with 5% Blotto, non-fat dry-milk (Santa Cruz Biotechnology, sc-2324) for 1h at room temperature. The membrane was washed several times in PBST (1X Phosphate Buffer Saline, containing 0.05% Tween 20) and incubated overnight at 4°C in appropriate primary antibodies. The membranes were probed with peroxidase conjugated appropriate secondary antibodies. The information of the antibodies used is provided in Table 1. For visualization of the bands, the membranes were incubated with Super Signal West Pico and Pierce West Femto maximum sensitivity chemiluminescent substrates (Thermo Scientific, Cat.#34080 and 34096) or Pierce ECL2 Western Blotting Substrate (Thermo Scientific, Cat.#80196) as per the manufacturer's instructions. The membranes were superimposed onto UltraCruz autoradiography films (Santa Cruz, Cat. # sc-201697) and the films were developed using Carestream GBX Developer (Cat.#1900984) and Fixer (Cat.#1902485) solutions purchased from Sigma. The blots were quantitated using imageJ software.

**Table 1:** List of antibodies used in this study.

**Antibody**	**Source**	**Catalog No**.
Anti-Oct4 antibody-ChIP grade	Abcam	ab19857
Nanog (1E6C4) Mouse mAb	Cell Signaling Technology	4893
Sox2 (L73B4) Mouse mAb	Cell Signaling Technology	4195
Monoclonal Anti-β-Tubulin Isotype III antibody produced in mouse	Sigma-Aldrich	T-5076
Purified anti-PAX-6	Biolegend	901301
OTX2 (D7Y3J) Rabbit mAb	Cell Signaling Technology	11943
Anti-Tbr1 Antibody	Millipore	AB2261
PARP Antibody	Cell Signaling Technology	9542
Caspase-3 Antibody	Cell Signaling Technology	9662
GAPDH (14C10) Rabbit mAb	Cell Signaling Technology	2118
Ki-67 (D2H10) Rabbit mAb (IHC Specific)	Cell Signaling Technology	9027
Anti-AKT1/2/3 antibody	Abcam	ab126811
Anti O-Linked N-Acetylglucosamine antibody [RL2]	Abcam	ab2739
Anti-pan-AKT (phosphor T308) antibody	Abcam	Ab38449
Anti-PCNA antibody	Abcam	Ab18197
β-*Actin* Antibody (C4)	Santa Cruz Biotechnology	sc-47778
Peroxidase AffiniPure Goat Anti-Mouse IgG, Fcγ Subclass 2b Specific	Jackson Immuno Research	115-035-207
Peroxidase AffiniPure Goat Anti-Mouse IgG (H+L)	Jackson Immuno Research	115-035-166
Peroxidase AffiniPure Goat Anti-Rabbit IgG (H+L)	Jackson Immuno Research	111-035-144
Peroxidase AffiniPure Rabbit Anti-Chicken IgY (IgG) (H+L)	Jackson Immuno Research	303-035-003
Anti-mouse IGg2b gamma chain (Alexa Fluor 488)-Rat monoclonal	Abcam	ab172326
Goat Anti-chicken IgY H&L (Alexa Fluor 488)	Abcam	ab150169
Goat Anti-Mouse IgG H&L (Alexa Fluor® 594) preadsorbed	Abcam	ab150120
Goat Anti-Mouse IgG H&L (Alexa Fluor® 488) preadsorbed	Abcam	ab150117
Goat Anti-Rabbit IgG H&L (Alexa Fluor® 594) preadsorbed	Abcam	ab150084

### RNA isolation and RT-PCR

Total RNA was isolated using MasterPure™ RNA Purification Kit from Epicentre (Cat.#MCR 85102) according to manufacturer's instructions. For cDNA synthesis, 1.0 ug of total RNA was reverse transcribed in a 20 ul reaction using SuperScript® VILO cDNA Synthesis Kit (Cat.# 11754050) as per manufacturer's instructions. Real time qRT-PCR was performed using SYBR® Green Real-Time PCR Master Mix (ThermoFisher Scientific) in a 10 or 20 ul reaction volume. The primer sequences used in the real time PCR reactions were obtained from Primer Bank (https://pga.mgh.harvard.edu/primerbank/index.html) and are listed in Table 2.

**Table 2:** Primer sequences used in this study.

**Primer name**	**Primer bank ID**	**5′-3′ sequence**
OCT4-Forward	4505967a2	GGGAGATTGATAACTGGTGTGTT
OCT4-Reverse	4505967a2	GTGTATATCCCAGGGTGATCCTC
NANOG-Forward	153945815c1	TTTGTGGGCCTGAAGAAAACT
NANOG-Reverse	153945815c1	TTTGTGGGCCTGAAGAAAACT
SOX2-Forward	325651854c3	TACAGCATGTCCTACTCGCAG
SOX2-Reverse	325651854c3	GAGGAAGAGGTAACCACAGGG
POLR2G-Forward	219879812c1	ATCTCCCTAGAGCACGAAATCC
POLR2G-Reverse	219879812c1	ACAAAGCCATACTTCCCTGTG
β-*ACTIN*-Forward	4501885a1	CATGTACGTTGCTATCCAGGC
β-*ACTIN*-Reverse	4501885a1	TCTTCATGAGGTAGTCAGTCAGGT
PAX6-Forward	189083679c1	TGGGCAGGTATTACGAGACTG
PAX6-Reverse	189083679c1	ACTCCCGCTTATACTGGGCTA
EMX2-Forward	164607120c1	CGGCACTCAGCTACGCTAAC
EMX2-Reverse	164607120c1	CAAGTCCGGGTTGGAGTAGAC
POU3F2-Forward	380254475c2	AAGCGGAAAAAGCGGACCT
POU3F2-Reverse	380254475c2	GTGTGGTGGAGTGTCCCTAC
OTX2-Forward	27436932c1	CAAAGTGAGACCTGCCAAAAAGA
OTX2-Reverse	27436932c1	TGGACAAGGGATCTGACAGTG
BDNF-Forward	219842299c2	TAACGGCGGCAGACAAAAAGA
BDNF-Reverse	219842299c2	TGCACTTGGTCTCGTAGAAGTAT
FOXG1-Forward	375151583c1	GAGCGACGACGTGTTCATC
FOXG1-Reverse	375151583c1	GCCGTTGTAACTCAAAGTGCTG
ST7-Forward	54112123c2	ATTAACACCGAAGTTCTACGTGG
ST7-Reverse	54112123c2	TGAATGAAGTTCCGTATTTGCGA
GRM4-Forward	378548207c1	GATTCAGCTTATGAGCAGGAGG
GRM4-Reverse	378548207c1	CTGGGTGCCATCTACAGGG
FOXP2-Forward	298566290c2	GCGTCAGGGACTCATCTCC
FOXP2-Reverse	298566290c2	GAGGTCTAGCCCTCCATGTTTA
COMT-Forward	205830450c2	GAAGGGGACAGTGCTACTGG
COMT-Reverse	205830450c2	CAGGAACGATTGGTAGTGTGTG
MECP2-Forward	160707949c1	TGACCGGGGACCCATGTAT
MECP2-Reverse	160707949c1	CTCCACTTTAGAGCGAAAGGC
GABRB2-Forward	124294884c2	GGGATGAACATTGACATTGCCA
GABRB2-Reverse	124294884c2	CTCCAGGCTTGTTGAAAGTACAT
APBA2-Forward	194353991c3	GAGGCCCAAGTCGCTGAAC
APBA2-Reverse	194353991c3	ACAGGTCCATTGAGATCAGAGC
OXTR-Forward	32307151c1	CTGCTACGGCCTTATCAGCTT
OXTR-Reverse	32307151c1	CGCTCCACATCTGCACGAA
RELN-Forward	223718142c1	CAACCCCACCTACTACGTTCC
RELN-Reverse	223718142c1	TCACCAGCAAGCCGTCAAAAA
CREBBP-Forward	119943103c1	CAACCCCAAAAGAGCCAAACT
CREBBP-Reverse	119943103c1	CCTCGTAGAAGCTCCGACAGT
Neurod1-Forward	380254474c1	ATGACCAAATCGTACAGCGAG
Neurod1-Reverse	380254474c1	GTTCATGGCTTCGAGGTCGT
DCX-Forward	306482598c1	TCCCGGATGAATGGGTTGC
DCX-Reverse	306482598c1	GCGTACACAATCCCCTTGAAGTA
EOMES-Forward	22538469c2	GTGCCCACGTCTACCTGTG
EOMES-Reverse	22538469c2	CCTGCCCTGTTTCGTAATGAT
CUX1-Forward	321400113c1	GAAGAACCAAGCCGAAACCAT
CUX1-Reverse	321400113c1	AGGCTCTGAACCTTATGCTCA
VGLUT1-Forward	221316691c3	CGACGACAGCCTTTTGTGGT
VGLUT1-Reverse	221316691c3	GCCGTAGACGTAGAAAACAGAG
PTEN-Forward	110224474c2	TTTGAAGACCATAACCCACCAC
PTEN-Reverse	110224474c2	ATTACACCAGTTCGTCCCTTTC
TBR1-Forward	22547231c3	TCTGAGCTTCGTCACAGTTTC
TBR1-Rverse	22547231c3	GCTGTTGTAGGCTCCGTTG

### Statistical analysis

Neural differentiation of hESCs, treated with PUGNAc/TMG was performed 3–4 times. All other experiments were performed in triplicates. Results shown are mean ± standard deviation (SD). Statistical analyses were made using GraphPad software (two-tailed unpaired Student's *t*-test) with asterisks representing differences being significant (^*^p ≤ 0.05, ^**^p ≤ 0.01, ^***^p ≤ 0.001).

## Ethics statement

All of the animal work has been evaluated and approved by animal ethics committee UAE University (ERA-2015-3210).

## Author contributions

SA, DV, and SP performed cell culture experiments, SP and DV performed qRT-PCR, SP performed western blotting, MA and SP performed immunocytochemistry and helped on manuscript preparation, AP and EM-B helped with the animal experiments, BE helped with experiment designing and data analysis and manuscript preparation, SA secured funds for this work, analyzed the final data and wrote the manuscript. All authors read and corrected the manuscript and approved its final content.

### Conflict of interest statement

The authors declare that the research was conducted in the absence of any commercial or financial relationships that could be construed as a potential conflict of interest.
