# Supplementary material for: Higher O-GlcNAc Levels Are Associated with Defects in Progenitor Proliferation and Premature Neuronal Differentiation during in-Vitro Human Embryonic Cortical Neurogenesis
Source: Front Cell Neurosci. 2017 Dec 21;11:415. doi: 10.3389/fncel.2017.00415 (PMC5742625; doi:10.3389/fncel.2017.00415)
Supplement: Supplementary file 1 [file Presentation1.PDF]

**Figure S1. Total O-GlcNac levels are elevated in the brains of pups from hyperglycemic rats during development.** Western blot analysis was performed for total O-GlcNAc from brain tissue lysates of pups from E16.5 and E18.5 developmental stages of hyperglycemic (STZ) and control (C) Wistar rats (left panel). Densitometric quantitation of blots was performed using ImageJ software (right panel). The expression of Gapdh was used for normalization. The data shown here are indicative of three biological replicates and are represented as mean ± standard deviation. * p≤0.05, **p≤0.01, ***p≤0.001 (two-tailed unpaired Student's t test).

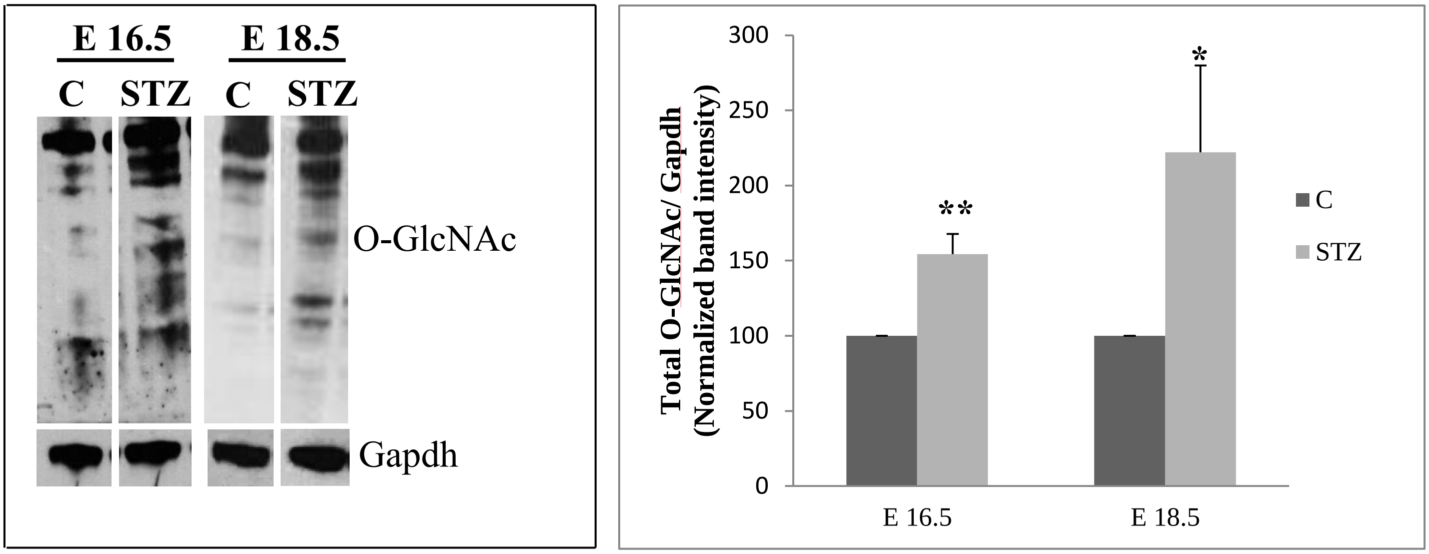

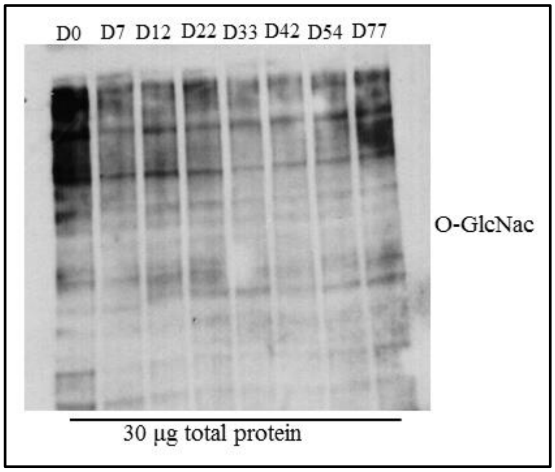


**Figure S2. Total O-GlcNac levels go down during cortical differentiation of H9 cells.** H9 cells were differentiated into cortical neurons and cell lysates were reserved from indicated stages of cortical differentiation. Western blot was performed on 30 μg of total protein for each sample to analyze the levels of total O-GlcNac using anti O-GlcNac antibody (RL2).

**Supplementary Method**

**Streptozotocin (STZ) treatment**

Gestational diabetes was induced by administration of a single bolus of streptozotocin (50 mg/kg given on day1 of pregnancy [1]. Rats were fed ad libitum with normal chow and water throughout the pregnancy. A 12h dark and light cycle was maintained. The pregnant rats were caged separately and monitored throughout the gestational period. On days E16.5 and E18.5, three controls and three STZ treated pregnant rats from both stages were euthanized by pentobarbital or urethane. Brains of 3-4 pups from each animal were pooled and stored at -80$^{0}$C for further analysis. All animal experiments were reviewed and approved by the Animal Ethics Committee of the UAE University.

**References**

[1]. Caluwaerts S, Holemars K, van Bree R, Verhaeghe J, Van Assche FA. Is low-dose streptozotocin in rats an adequate model for gestational diabetes mellitus? J. Soc. Gynecol Inves. 2003; 10: 216-21.
